# Microfragmented abdominal adipose tissue‐derived stem cells from knee osteoarthritis patients aged 29–65 years demonstrate in vitro stemness and low levels of cellular senescence

**DOI:** 10.1002/jeo2.12056

**Published:** 2024-06-21

**Authors:** Freja Aabæk Hammer, Per Hölmich, Jan O. Nehlin, Kilian Vomstein, Lars Blønd, Lisbet Rosenkrantz Hölmich, Kristoffer Weisskirchner Barfod, Jasmin Bagge

**Affiliations:** ^1^ Sports Orthopedic Research Center—Copenhagen (SORC‐C), Department of Orthopedic Surgery Copenhagen University Hospital—Amager and Hvidovre Hvidovre Denmark; ^2^ Department of Clinical Research Copenhagen University Hospital—Amager and Hvidovre Hvidovre Denmark; ^3^ Department of Obstetrics and Gynecology, The Fertility Clinic Copenhagen University Hospital—Hvidovre Hvidovre Denmark; ^4^ Department of Orthopedic Surgery Zealand University Hospital—Køge Køge Denmark; ^5^ Department of Plastic Surgery Copenhagen University Hospital—Herlev and Gentofte Herlev Denmark

**Keywords:** adipose tissue‐derived stem cells, ageing, cellular senescence, microfragmentation, osteoarthritis

## Abstract

**Purpose:**

To investigate the level of cellular senescence in stem cells derived from microfragmented abdominal adipose tissue harvested from patients with knee osteoarthritis (OA).

**Methods:**

Stem cells harvested from microfragmented abdominal adipose tissue from 20 patients with knee OA, aged 29–65 years (mean = 49.8, SD = 9.58), were analysed as a function of patient age and compared with control cells exhibiting signs of cellular senescence. Steady‐state mRNA levels of a panel of genes associated with senescence were measured by qPCR. Intracellular senescence‐associated proteins p16 and p21, and senescence‐associated β‐galactosidase activity were measured by flow cytometry. Cellular proliferation was assessed using a 5‐ethynyl‐2′‐deoxyuridine proliferation assay. Stemness was assessed by stem cell surface markers using flow cytometry and the capacity to undergo adipogenic and osteogenic differentiation in vitro.

**Results:**

No correlation was found between cellular senescence levels of the microfragmented adipose tissue‐derived stem cells and patient age for any of the standard assays used to quantify senescence. The level of cellular senescence was generally low across all senescence‐associated assays compared to the positive senescence control. Stemness was verified for all samples. An increased capacity to undergo adipogenic differentiation was shown with increasing patient age (*p* = 0.02). No effect of patient age was found for osteogenic differentiation.

**Conclusions:**

Autologous microfragmented adipose tissue‐derived stem cells may be used in clinical trials of knee OA of patients aged 29–65 years, at least until passage 4, as they show stemness potential and negligible senescence in vitro.

**Level of Evidence:**

Not applicable.

**Abbreviations**
ADSC: adipose tissue‐derived stem cell
ASC: adventitial stem cell
AT: adipose tissue
BSA: bovine serum albumin
CD: cluster of differentiation
CDKN1A: cyclin‐dependent kinase inhibitor 1A
CDKN2A: cyclin‐dependent kinase inhibitor 2A
cDNA: complementary deoxyribonucleic acid
CO_2_
: carbon dioxide
Ct: cycle threshold
CXCL8: C‐X‐C motif chemokine ligand 8
DAPI: 4′,6‐diamidino‐2‐phenylindole dihydrochloride
DMSO: dimethyl sulfoxide
DNA: deoxyribonucleic acid
dPBS: Dulbecco's phosphate‐buffered saline
dsDNase: double stranded deoxyribonuclease
EDTA: Ethylenediaminetetraacetic acid
EDU: 5‐ethyl‐2′‐deoxyuridine
FACS: fluorescence activated cell sorting
FAM: fluorescein amidite
FBS: fetal bovine serum
FITC: fluorescein isothiocyanate
FSC‐A/H: forward scatter‐area/height
GAPDH: glyceraldehyde‐3‐phosphate dehydrogenase
GLB1: galactosidase β1
GUSB: glucuronidase‐β
H_2_O_2_
: hydrogen peroxide
IgG_1_κ: immunoglobulin subclass 1 light chain type κ
IL6: interleukin 6
IL8: interleukin 8/protein encoded by CXCL8
MF: microfragmented
mRNA: messenger ribonucleic acid
OA: osteoarthritis
p16: protein encoded by CDKN2A
p21: protein encoded by CDKN1A
p53: protein encoded by TP53
qPCR: quantitative polymerase chain reaction
Q–Q plot: quantile–quantile plot
RNA: ribonucleic acid
RQ: relative expression
SASP: senescence‐associated secretory phenotype
SA‐β‐GAL: senescence‐associated β‐galactosidase
SD: standard deviation
SORC‐C: Sports Orthopaedic Research Center—Copenhagen
SSC‐A/H: side scatter area/height
T75: 75 cm^2^ tissue culture flask
TP: transitional pericytes
TP53: tumour protein 53
Yo.: year old

## BACKGROUND

Osteoarthritis (OA) is a progressive, degenerative joint disorder and one of the primary causes of disability among the adult population [18] with the knee being the most affected joint [14]. The rising incidence of OA with age, coupled with the rapidly increasing global ageing population, emphasizes the growing importance of this disease. Yet, conventional treatments are palliative in nature and do not have the ability to regenerate the affected joint [6, 29].

Treatment of OA with multipotent stem cells derived from microfragmented (MF) adipose tissue (AT) has shown promising results with improved patient‐reported outcome measurements, reduced disease progression, and enhanced cartilage regeneration on imaging and synovial fluid analysis [6, 23, 46]. Microfragmentation is a mechanical and non‐enzymatic processing of AT that preserves the tissue microarchitecture and takes advantage of both active adipose tissue‐derived stem cells (ADSCs) and their environment in a one‐step surgical procedure [17, 39]. An advantage of ADSCs is that they can be easily and gently harvested by liposuctions, which yields a relatively high quantity of stem cells as a heterogeneous population [2, 32]. Moreover, microfragmentation of AT has been shown to provide a pericyte‐enriched stem cell population, which could have clinical benefits [48].

ADSCs have been shown to function through self‐renewal, migration and homing to the site of injury, and differentiation into osteocytes and chondrocytes [1, 39, 50]. However, their primary function is believed to involve paracrine signalling, which helps counteract degenerative processes and decrease pain by modulating inflammation, inhibiting fibrosis and apoptosis, and promoting the activation of local progenitor cells to facilitate healing [39, 42].

Even though treatment of OA with ADSCs is promising, there is an inconsistency between studies as every study has its own setup including way of administration, number of ADSCs applied, and patient age [37]. Multiple studies have shown a decline in stem cell potential with increasing patient age, thereby suggesting it is a potential contributing factor to a poorer treatment outcome [43]. Studies have reported a decline in the number of ADSCs, population doublings, and osteogenic and chondrogenic differentiation performance of ADSCs with increasing donor age [3, 10]. This is highly important, particularly because autologous cell therapies are extensively used in elderly OA patients.

These changes may be explained by an increase in senescent stem cells in aged patients, which previously has been documented on enzymatically processed ADSCs commonly used for regenerative therapies [10]. Cellular senescence is a state of irreversible cell cycle arrest that serves as a protective mechanism for normal somatic cells exposed to an accumulation of stress factors. It ensures a limited proliferative capacity of these cells to protect them from becoming cancerous [12, 30]. As a result, senescent cells are unable to proliferate or be expanded to achieve higher cell numbers, which is needed for certain therapeutical applications. Senescent cells also secrete distinct paracrine and autocrine signalling factors as part of their senescence‐associated secretory profile (SASP), which can inhibit the function of functional stem cells and contribute to the development of chronic inflammation [22]. In addition, senescent stem cells have lost their ability to undergo chondrogenic and osteogenic differentiation [19].

High levels of cellular senescence may thus diminish treatment outcome, due to the lost proliferative and differentiation capacity, as well as the secretion of inhibitory SASP factors that affect the surrounding functional cells [51]. The level of cellular senescence, amongst MF‐ADSCs remains to be investigated across multiple levels.

The aim of this study was to examine the level of cellular senescence in MF‐ADSCs from abdominal AT of knee OA patients as a function of patient age by (1) measuring steady‐state mRNA levels of senescence‐associated biomarkers, (2) identifying cells with intracellular senescence‐associated proteins p16 and p21, (3) cells with senescence‐associated beta‐galactosidase (SA‐β‐GAL) activity and (4) non‐proliferating cells, in samples from OA patients of different ages. Furthermore, stemness was assessed to validate MF AT as having the potential to be used in clinical stem cell trials of knee OA.

## METHODS

### Patient inclusion

MF‐ADSCs from 20 knee OA patients were included in an ongoing randomized controlled trial examining the treatment of knee OA with autologous MF AT (ClinicalTrials.gov Identifier: NCT03771989) [34]. Patients included had to suffer from pain and functional impairment due to an OA Kellgren–Lawrence Grades 2–3 in the tibiofemoral joint [6]. A full list of inclusion and exclusion criteria can be found at (ClinicalTrials.gov Identifier: NCT03771989) [34].

### Harvest of MF‐ADSCs

Subcutaneous abdominal AT was harvested by lipoaspiration under local analgesia and sterile conditions, followed immediately by microfragmentation to reduce the size of the AT clusters and release the MF‐ADSCs while removing blood components as described by Mikkelsen et al. [34].

Seven of the MF lipoaspirates were directly cryopreserved for 46–150 days as described in Bagge et al. [4] until the start of tissue explant culture isolation and cultivation. The remaining samples (*n* = 13) were isolated by tissue explant cultivation immediately after harvesting according to the procedure described in Bagge et al. [4].

### Cultivation of MF‐ADSCs

MF‐ADSCs were grown as monolayers in 75 cm^2^ Tissue culture flasks (T75 flasks, TPP, Cat# 90076) with 11 mL AT expansion medium containing Dulbecco's Modified Eagle Medium (Gibco, Cat# 11580586, with 1 g/L glucose, phenol red, GlutaMAX and pyruvate), 10% heat‐inactivated fetal bovine serum (FBS, Gibco, Cat# 11573397) and 1% Penicillin‐Streptomycin (Gibco, Cat# 11528876). The cells were kept under humidified conditions at 37°C and 5% CO_2_ and medium change was performed two to three times per week.

MF‐ADSCs were trypsinized for further passaging with 1.5 mL 0.25% Trypsin‐Ethylenediaminetetraacetic acid (EDTA, Gibco, Cat# 11560626) per T75 flask when reaching approximately 70% confluency. The number of live cells in the suspension was counted from an aliquot using trypan blue staining (Gibco, Cat# 11538886) and a Fast‐Read® 102 cell counter (VWR, Cat#630‐1893). The cell suspension was then evenly distributed into new T75 flasks with a seeding density of 5–7.5 × 10^5^ cells/T75 flask.

### Storage of MF‐ADSCs: Freezing and thawing

In order to handle 20 different cell lines, cultivation was performed in sequential stages for logistical convenience.

Expanded MF‐ADSCs were cryopreserved when reaching approximately 70% confluency at passage 1. Cryopreserved ADSCs have been shown to be functionally equivalent to ADSCs obtained from fresh AT [11]. The cells were cryopreserved at a concentration of 1 × 10^6^ cells/mL in cryomedium containing 90% FBS and 10% dimethyl sulfoxide (DMSO, Sigma‐Aldrich, Cat# D2650‐100ML) [24]. Volumes of 1 mL cell suspension were transferred to cryogenic tubes (Thermo Fisher Scientific, Cat# 10344691) to be kept at −80°C overnight in a slow‐freeze BioCision CoolCell LX before being transferred for long‐term storage at −150°C.

To initiate the thawing process, cryogenic tubes containing frozen MF‐ADSCs were retrieved from the −150°C freezer and transported on dry ice. The vials were carefully dipped in lukewarm water. When thawed, the cells were immediately transferred to a 15 mL conical tube (Thermo Fisher Scientific, Cat# 352097) and 1 mL of 37°C prewarmed AT expansion medium was added dropwise while stirring. An additional 5 mL AT expansion medium was slowly added. The cells were centrifuged at 500*g* for 5 min and washed with Dulbecco's phosphate‐buffered saline (dPBS; Gibco, Cat# 15326239) to remove any remains of the cell toxic DMSO. Cells from one cryogenic tube were resuspended in AT expansion medium and seeded into one T75 flask. For further cell expansion, seeding densities of 500,000–750,000 cells/T75 were used to avoid low seeding densities, which may lead to increased cell divisions before passaging and consequently precipitate the onset of cellular senescence. All assays were performed at passage 4.

### Immunophenotyping of MF‐ADSCs by flow cytometry

**Figure 1:**
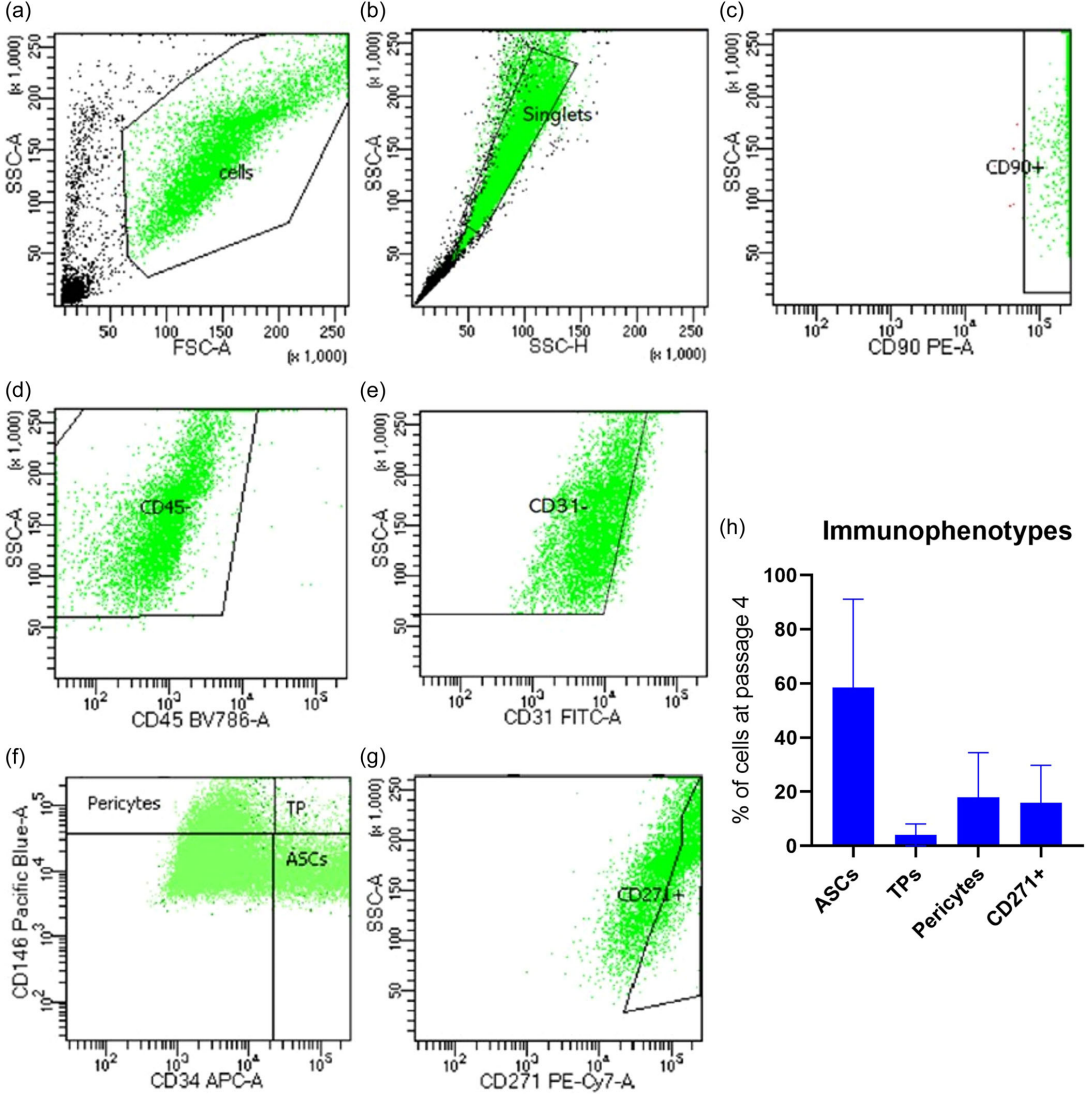
Gating strategy for the identification of MF‐ADSC subtypes using multicolour flow cytometry at passage 4. (a) Selection of cells of interest by removal of debris based on SSC‐A and FSC‐A. (b) Selection of singlet cells based on SSC‐A and SSC‐H. (c) Selection of CD90+ cells. (d) Selection of CD45− cells. (e) Selection of CD31‐ cells. (f) Adventitial stem cells (ASCs) were identified as CD31−CD34+CD45−CD90+CD146−, pericytes as CD31−CD34−CD45−CD90+CD146+, and transitional pericytes (TPs) as CD31−CD34+CD45−CD90+CD146+. (g) Selection of CD271+ cells identified as CD31−CD45−CD90+CD271+. (h) Immunophenotypes present in MF AT. Bar chart showing the percentage (%) of ASCs, TPs, pericytes, and CD271+ stem cells. Bar charts show mean and standard deviation. ADSC, adipose tissue‐derived stem cell; AT, adipose tissue; FSC‐A, forward scatter‐area; MF, microfragmented; SSC‐A, sideward scatter‐area.

Flow cytometry was used to analyse the immunophenotype of the MF‐ADSCs at passage 4 according to the manufacturer's protocol using multiple anti‐human fluorescent primary antibodies; CD31‐FITC, CD34‐APC, CD45‐BV786, CD90‐PE, CD146‐BV421 and CD271‐PE‐CyTM7 (BD Biosciences, Horizon subtype, mouse IgG_1_κ isotype) and analysed on a flow cytometer (BD LSR Fortessa with BD FACSDiva 8.0.3 software) as described by Bagge et al. [4]. Flow cytometry data was analysed using BD FACSDiva Software 8.0.3 (Figure 1a–g).

### Adipogenic and osteogenic in vitro differentiation of MF‐ADSCs

MF‐ADSCs were trypsinized from passages 3 to 4 and seeded onto two 6‐well plates (NUNC, Thermo Fisher Scientific, Cat# 10469282) with a seeding density of 3000 cells/cm^2^. The cells were grown in 2 mL AT expansion medium/well until reaching ~90% confluency. Cells in three wells of one plate were induced to undergo adipogenic differentiation for 14 days, while cells in three wells of the second plate were induced to undergo osteogenic differentiation for 21 days. The remaining wells were kept as non‐induced controls and grown in AT expansion medium throughout the entire 14 and 21 days of the differentiation protocols, respectively. The cells were quantitatively stained with Oil‐Red‐O (Sigma‐Aldrich, Cat# O0625) to assess adipogenesis and Alizarin Red S (ARed‐Q, ScienCell, Cat# 8678) to assess osteogenesis, as described by Bagge et al. [4].

### Analysis of cellular senescence in MF‐ADSCs

As positive controls, MF‐ADSCs were induced to undergo cellular senescence, following two different induction methods.

First, MF‐ADSCs were passaged in T75 flasks at a very low seeding density to induce replicative senescence over a period of 7 months. The AT expansion medium was changed with increasingly longer time intervals, starting at two times a week and ending at once per month.

A second induction method using H_2_O_2_ to make the cells undergo stress‐induced premature senescence was used as a positive senescence control for the intracellular senescence‐associated p16 + p21 proteins assay. A pilot study was conducted based on Duan et al. and Wang et al. [16, 49] to determine the optimal seeding density and concentration of H_2_O_2_ for induction of cellular senescence of MF‐ADSCs. Data from the pilot study showed an optimal H_2_O_2_ senescence induction of MF‐ADSCs using the following procedure: two T75 flasks with 50% confluency were spiked with 200 μM H_2_O_2_ (Thermo Fisher Scientific, Cat# 033323.AD) in AT expansion medium and incubated for 2 h at 37°C and 5% CO_2_. Then, the medium was replaced by AT expansion medium containing 10 μM H_2_O_2_ for maintenance. Medium change with 10 μM H_2_O_2_ was performed every 2 days. The cells were examined by light microscopy to follow morphological changes every 2 days. Senescence‐associated assays were performed on the cells 8 days after the first spiking with H_2_O_2_.

### Quantification of cellular senescence by qPCR

MF‐ADSCs were harvested at passage 4 when reaching approximately 70% confluency in a T75 flask. The medium was aspirated, and the cells were washed two times with dPBS. Next, the cells were flushed with 1 mL Qiazol® lysis reagent (Qiagen, Cat# 79306, Guanidinium lysis reagent) to lyse the cells and extract the RNA, and the suspension was transferred to a cryogenic tube. The tube was immediately snap‐frozen in liquid nitrogen and stored at −80°C prior to RNA isolation.

The samples were thawed on ice and 1 mL of Qiazol® was added per cryogenic tube. Total RNA was isolated using the spin column‐based Qiagen RNeasy Mini Kit® (Qiagen, Cat# 74104) with modifications as described by Mienaltowski et al. [33]. Briefly, the suspension was evenly distributed into four microcentrifuge tubes. Next, 200 μL chloroform (Thermo Fisher Scientific, Cat# 032614.K2) was carefully added on the top, and the tubes were vortexed, followed by 2.5 min of incubation at room temperature. Subsequently, the tubes were centrifuged at 12,000*g* for 20 min. Thereafter, the aqueous top phase was transferred and diluted 1:1 with 70% ethanol in nuclease‐free water (Invitrogen, Cat# AM9938) into new microcentrifuge tubes. The suspensions were added to the top of an RNeasy spin column, followed by centrifugation at 8000*g* for 20 s. Then, 700 μL RWI buffer was added and the spin column was centrifuged, followed by addition of 500 μL RPE buffer to the column, which was repeated two times. The column was transferred to a new collection tube and centrifuged at 18,213*g* for 1 min. RNA was released from the columns by adding 20 μL 70°C nuclease‐free water, followed by centrifugation at 8000*g* for 1 min. This step was repeated once more. The RNA yield was measured using a Nanodrop 1000 spectrophotometer (Thermo Fisher Scientific) and a Qubit® 3.0 fluorometer (Thermo Fisher Scientific). Nuclease‐free water (Invitrogen, Cat# AM9938) was added to a final volume of 180 μL. Next, 20 μL 3 M Sodium Acetate pH 5.2 (Thermo Fisher Scientific, Cat# R1181) was added and the tube was vortexed before adding 500 μL ice‐cold 100% ethanol (Thermo Fisher Scientific, Cat# 445730010). The tube was stored at −80°C for a minimum of 2 h before proceeding with ethanol precipitation. Tubes were thawed on ice and centrifuged at 18,000*g* for 25 min at 4°C. The pellet was dried and 10−20 μL nuclease‐free water was added before measuring the RNA yield. Nuclease‐free water was added to a final concentration of 100 ng/μL RNA and the tube was stored at −80°C prior to cDNA conversion.

All purified RNA samples were measured with a Nanodrop at 260/280 and 260/230 nm wavelength ratios yielding values close to 2, thus meeting RNA quality thresholds. Qubit RNA concentration measurements ranged between 224 and 4480 ng/μL using the Qubit RNA Broad Range assay kit (Thermo Fisher Scientific, Cat# Q10210).

Removal of potential genomic DNA contamination and first strand cDNA synthesis was performed using the Maxima First Strand cDNA Synthesis Kit for qPCR, with dsDNase (Thermo Fisher Scientific, Cat# K1671) as per manufacturer's protocol. Samples were diluted to 20 ng/μL cDNA and stored at −80°C.

Steady‐state mRNA levels were quantified with commercially available, validated human‐specific TaqMan® primer‐probe sets (Thermo Fisher Scientific, Cat# 4331182, FAM‐dye labelled MGB probes) (Table 1). A panel of six biomarkers were selected for the senescence‐associated gene expression analysis, together with two endogenous controls (GUSB and GAPDH). The endogenous controls were tested against all samples. Using NormFinder software [37], GUSB was determined to have the most uniform performance across all samples (data not shown) and was thus used for later analysis. The expression of all biomarkers was tested against a positive sample from MF‐ADSCs induced to undergo replicative senescence. Negative controls of nuclease‐free water, all components minus template, and minus master mix were included on all qPCR plates. All samples and controls were run in duplicates using the TaqMan™ Fast Advanced Master Mix (Thermo Fisher Scientific, Cat# 4444556) kit according to the manufacturer's protocol. qPCR reactions were conducted in a 96‐well PCR plate (Life Science, Roche, Cat# 04729692001) using the Roche LightCycler® 96 System. Data were analysed using the LightCycler® 96 software version 1.1.0.1320, where cycle threshold (Ct) values were calculated. All targets demonstrated amplification efficiencies close to 2, with the exception of the negative controls which did not show any amplification. This calculation was made using a standard curve generated from serial dilutions of the positive control of replicative senescent MF‐ADSCs when analysed with the LightCycler® 96 software (data not shown). ΔCt values were calculated for each sample by subtracting the corresponding Ct value of the endogenous control (GUSB). The ΔCt of the positive senescence control was used as a calibrator to calculate ΔΔCt values. The relative expression (RQ) of the gene targets was calculated by the formula RQ = 2−ΔΔCt. As this is an exponential function, ln(RQ) values were calculated to linearize the data again for statistics and visualization of data [31].

**Table 1:** Overview of TaqMan® primer‐probes sets used in qPCR reactions.

Gene ID	Gene name	ThermoFisher Assay ID
CDKN2A	p16, cyclin‐dependent kinase inhibitor 2A	Hs00923894_m1
CDKN1A	p21, cyclin‐dependent kinase inhibitor 1A	Hs00355782_m1
TP53	p53, tumour protein 53	Hs01034249_m1
GLB1	Galactosidase β1	Hs01035168_m1
IL6	Interleukin‐6	Hs00174131_m1
CXCL8	IL8 C‐X‐C motif chemokine ligand 8	Hs00174103_m1
GUSB	Glucuronidase‐β (endogenous control)	Hs00939627_m1
GAPDH	Glyceraldehyde‐3‐phosphate dehydrogenase (endogenous control)	Hs02786624_g1

### Quantification of cellular senescence using flow cytometry: Measurement of protein levels of intracellular p16 and p21 senescence biomarkers

**Figure 2:**
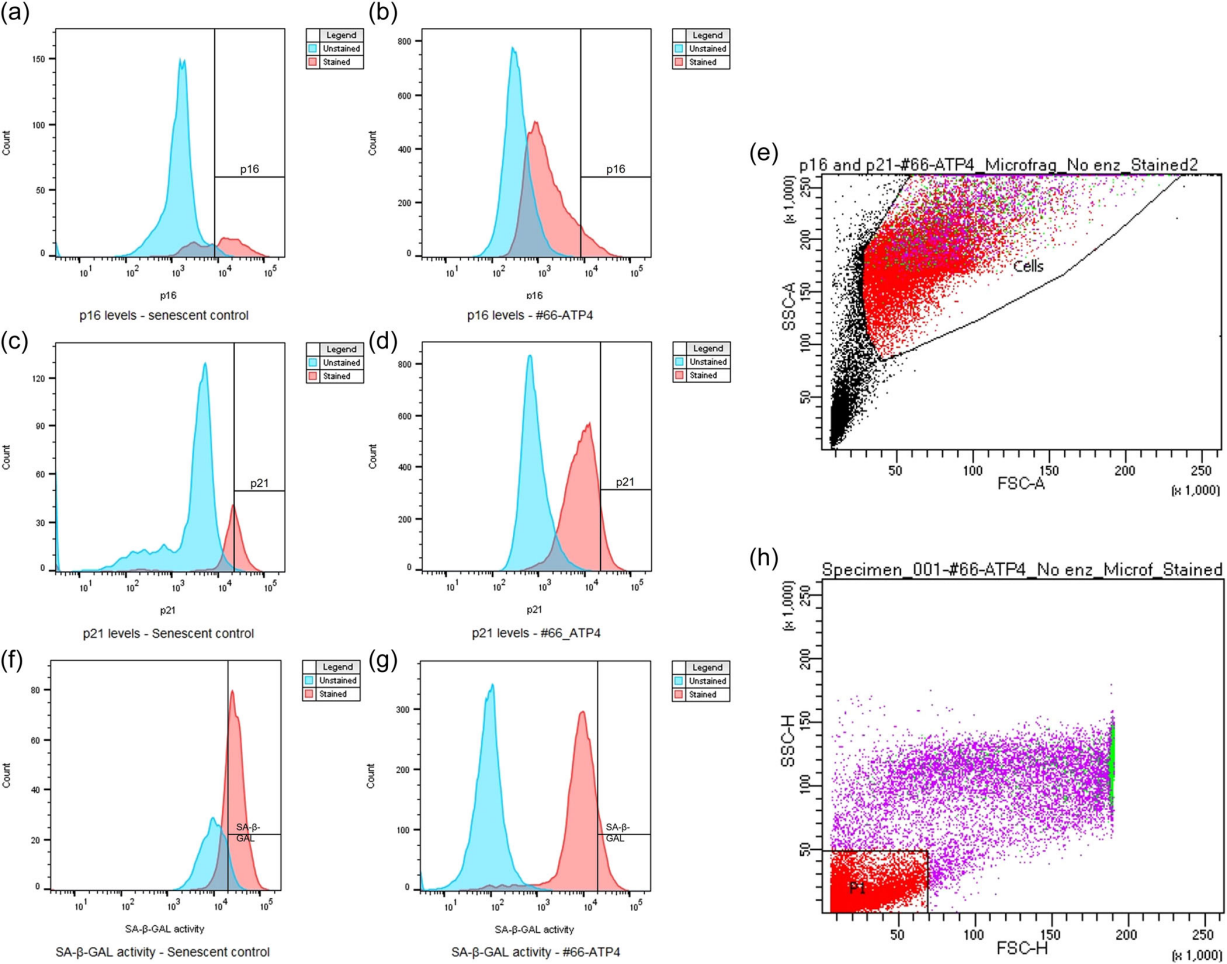
Flow cytometry gating strategy for (a–e) the intracellular senescence markers p16 and p21 after 1 h of labelling MF‐ADSCs, at passage 4, with p16 antibody Alexa fluor® 647 conjugate and p21 antibody Alexa fluor® 488 conjugate. (a, c) MF‐ADSCs induced to undergo stress‐induced premature senescence for 8 days using 200 μM H_2_O_2_ for 2 h, followed by 10 μM H_2_O_2_ for maintenance (positive control used for gating). (b, d) A representative (untreated) sample of MF‐ADSCs at passage 4. Cells to the right of the vertical lines were determined to be p16 and p21 positive. (e) Selection of cells of interest by removal of debris based on SSC‐A and FSC‐A. Purple dots indicated cells positive for p16 and green dots indicated cells positive for p21. Gating strategy for (f–h) the SA‐β‐GAL activity assay after 2 h of spiking passage 4 MF‐ADSCs with senescence dye at pH 6. (f) MF‐ADSCs induced to undergo replicative senescence (positive control used for gating). (g) A representative sample of (untreated) MF‐ADSCs at passage 4. Cells to the right of the vertical line were determined to be SA‐β‐GAL positive. (h) Selection of cells of interest by removal of debris based on SSC‐H and FSC‐H. Green dots indicated cells with SA‐β‐GAL activity. ADSC, adipose tissue‐derived stem cell; FSC‐A, forward scatter‐area; MF, microfragmented; SSC‐A, sideward scatter‐area.

MF‐ADSCs expressing intracellular senescence‐associated proteins p16 and p21 were quantified by flow cytometry according to a modified version of the manufacturer's protocol (Cell Signaling Technology, Cat# 43161). At 70% confluency, passage 4 cells grown in a T75 flask were trypsinized and harvested. The cell pellet was resuspended and fixed in 100 μL 37°C prewarmed 4% formaldehyde (Thermo Fisher Scientific, Cat# 11586711, methanol free) in dPBS for 15 min at room temperature in a 15 mL conical tube. The cells were washed with dPBS, and the pellet was resuspended in 1 mL dPBS and incubated at 4°C for 10 min. The cells were permeabilized by adding 9 mL ice‐cold 100% methanol (Sigma‐Aldrich, Cat# 322415) dropwise while stirring and incubated for 15 min on ice. The cell suspension was pelleted by centrifugation at 500*g* for 5 min and diluted in dPBS to ensure even distribution across two FACS tubes (Thermo Fisher Scientific, Cat# 10585801) and subsequently pelleted again. In one of the tubes, a total volume of 96 μL antibody dilution buffer containing 0.5% bovine serum albumin (BSA, Sigma‐Aldrich, Cat# A7906‐50G) in dPBS was added to block unspecific binding. Additionally, anti‐human p16 antibody Alexa flour® 647 conjugate (Cell Signaling Technology, Cat# 43161S) and anti‐human p21 antibody Alexa flour® 488 conjugate (Cell Signaling Technology, Cat# 5487S) were added in a 1:50 dilution. For the unstained negative control, the pellet was diluted in 100 μL antibody dilution buffer with no antibodies added. The stained and unstained samples were vortexed followed by incubation for 1 h at room temperature, protected from light. The samples were washed with 0.5 mL dPBS, and the pellets were resuspended in 350 μL antibody dilution buffer and kept protected from light before being immediately analysed on a BD LSR Fortessa flow cytometer (BD Biosciences) recording up to 30,000 events. Cells were sequentially gated based on forward scatter (FSC) and sideward scatter (SSC) parameters to visualize cell size and granularity. By gating cells with similar SSC‐area (‐A) and SSC‐height (‐H) values, non‐single cells, such as aggregates or debris, were excluded (Figure 2e). MF‐ADSCs exposed to H_2_O_2_ to induce stress‐induced premature senescence were used as a positive control to account for autofluorescence when gating and to identify p16^+^ and p21^+^ fractions used for gating. The non‐stained samples were used to optimize the gating strategy (Figure 2a–d). BD FACSDiva CS&T Research Beads (BD Biosciences) were used to check the performance of the flow cytometer prior to each assay. Analysis of flow cytometry data was performed using BD FACSDiva Software 8.0.3.

### Quantification of cellular senescence using flow cytometry: Measurement of SA‐β‐GAL activity

The SA‐β‐GAL activity of the MF‐ADSCs was quantitatively assessed using an SA‐β‐GAL activity assay kit for flow cytometry modified according to the manufacturer's protocol (Abcam, Cat# ab228562). At 70% confluency, passage 3 cells cultured in T75 flasks were split to passage 4 and transferred into three wells of a 24‐well plate (Thermo Fisher Scientific, Cat# 142475) with a seeding density of 1 × 10^5^ cells/well and 0.5 mL AT expansion medium/well. After 48 h of incubation at 37°C and 5% CO_2_, the medium was removed and 0.5 mL fresh AT expansion medium containing 1.5 μL Senescence dye from the kit was added to two of the wells. The dye incorporates a fluorogenic SA‐β‐GAL substrate (beta‐d‐galactopyranoside) which upon enzymatic cleavage generates a strong green fluorescent signal (excitation: 490 nm, emission: 514 nm). The remaining well was left without dye, constituting an unstained negative control. After incubation for 2 hours at 37°C and 5% CO_2_, the medium was removed, and the cells were washed twice with 0.5 mL wash buffer from the kit (pH 6). Cells were collected in FACS tubes by trypsinization and resuspended in wash buffer. The cells were centrifuged at 500*g* for 5 min. The pelleted cells were then resuspended in 350 μL wash buffer and kept protected from light before being immediately analysed on a BD LSR Fortessa flow cytometer recording 10–30,000 events. Cells were sequentially gated based on FSC and SSC parameters to visualize cell size and granularity. By gating cells with similar SSC‐H and SSC‐H values, non‐single cells, such as aggregates or debris, were excluded (Figure 2h). MF‐ADSCs induced to undergo replicative senescence were used as a positive control to account for autofluorescence when gating and to identify cells with SA‐β‐GAL activity. The non‐stained samples were used to optimize the gating strategy (Figure 2f,g). The results from the stained sample duplicates were averaged for further analysis.

### Determination of MF‐ADSC cellular proliferation

The ratio of proliferating MF‐ADSCs was quantified by determining the levels of incorporated 5‐ethyl‐2′‐deoxyuridine (EdU) [40]. EdU is a thymidine analogue that is integrated into the DNA when the cell goes through the S phase of the cell cycle. Fluorescently labelled EdU enables visualization of proliferating cells using Click‐IT chemistry and fluorescence microscopy, as described by the manufacturer using the Click‐IT Plus Alexa Fluor 594 EdU Imaging KIT® (Life Technologies, Cat# 10639). Briefly, passage 3 cells at 70% confluency grown in T75 flasks were split to passage 4. The cells were cultured in seven wells of a 24‐well plate with a seeding density of 2.5 × 10^4^ cells/well and with 0.5 mL AT expansion medium/well. After 48 h of incubation, 1.5 mL 5 μM EdU, prewarmed at 37°C in AT expansion medium was added to spike six of the wells. The last well served as the non‐EdU‐spiked negative control and was kept in AT expansion medium solely. After 24 h of incubation at 37°C and 5% CO_2,_ the cells were fixed with 37°C 4% formaldehyde in dPBS for 15 min. The cells were washed two times with 0.5 mL 3% BSA in dPBS. Next, 0.5 mL 0.5% Triton‐X‐100 (Sigma‐Aldrich, Cat# T9284‐100mL) in dPBS was added to permeabilize the cells for 20 min. Then, 0.5 mL 3% BSA in dPBS was used to wash the cells two times. To each well, 250 μL EdU Click‐iT Plus reaction cocktail was added, containing 1× Click‐iT reaction buffer, Copper protectant, Alexa Fluor picolyl azide, and 1× Reaction buffer additive. The cells were incubated for 30 min. The cells were washed with 0.5 mL 3% BSA in dPBS followed by a second wash with 0.5 mL dPBS. Subsequently, 300 μL 4′,6‐diamidino‐2‐phenylindole dihydrochloride (DAPI, Thermo Fisher Scientific, Cat# 10184322) in dPBS was added per well to counterstain all nuclei at a concentration of 1 µg/mL for 15 min. Excess dye was removed by washing with dPBS. The fluorophore staining cocktail was freshly prepared for each assay and the cells were subsequently incubated at room temperature, protected from light, during the staining procedure. Images were captured at 20× magnification from three non‐overlapping fields of each well using a fluorescence microscope Zeiss Axio Observer.Z1/7 with DAPI (exposure time 27–54 min) and Alexa 594 (exposure time 16–30 min) filters. The NIH ImageJ software version 1.53t was used to automatically count the total number of cells (DAPI stained) as well as the number of proliferating cells (Alexa 594 stained). Subsequent manual counting of each image was performed in ImageJ to verify the automated counting. The ratio of proliferating cells was determined by calculating the percentage of EdU‐labelled nuclei relative to the total number of cell nuclei in each image. The proliferation percentage was calculated by averaging the values obtained from the three images per well. Finally, the average for all six technical replicates per sample was averaged. Replicative senescent MF‐ADSCs were used as a correlative control.

### Statistical analyses

Statistical analyses were conducted to examine the concentration of Oil‐Red‐O and Alizarin Red S, as well as the senescence‐associated assays. Linear regression was employed as a function of patient age for these analyses. A Wilcoxon matched‐pair signed mark test was performed to compare induced and non‐induced samples for adipogenic and osteogenic differentiation as the data did not exhibit a normal distribution. Normality of data was assessed by QQ‐plots and Shapiro–Wilk tests. *p* Values <0.05 were considered statistically significant. All statistical analysis and graphical presentations were made in GraphPad Prism version 9.4.1.

## RESULTS

Levels of cellular senescence were assessed quantitatively at gene expression‐, protein‐, protein activity‐, and cellular functionality levels.

MF‐ADSCs from all 20 patients (age 29–65 years, mean: 49.8, SD: 9.58, 9 males and 11 females) were able to be expanded to ~70% confluency from passages 2–4.

### MF‐ADSC are multipotent and capable of undergoing adipogenic and osteogenic differentiation in vitro

Stemness was verified by multicolour flow cytometry for all 20 samples. Multiple types of stem cells were identified. These included adventitial stem cells (ASCs) (CD31−CD34+CD45−CD90+CD146−), transitional pericytes (CD31−CD34+CD45−CD90+CD146+), pericytes (CD31−CD34−CD45−CD90+CD146+), and CD271+ stem cells (CD31−CD45−CD90+CD271+) (Figure 1h).

**Figure 3:**
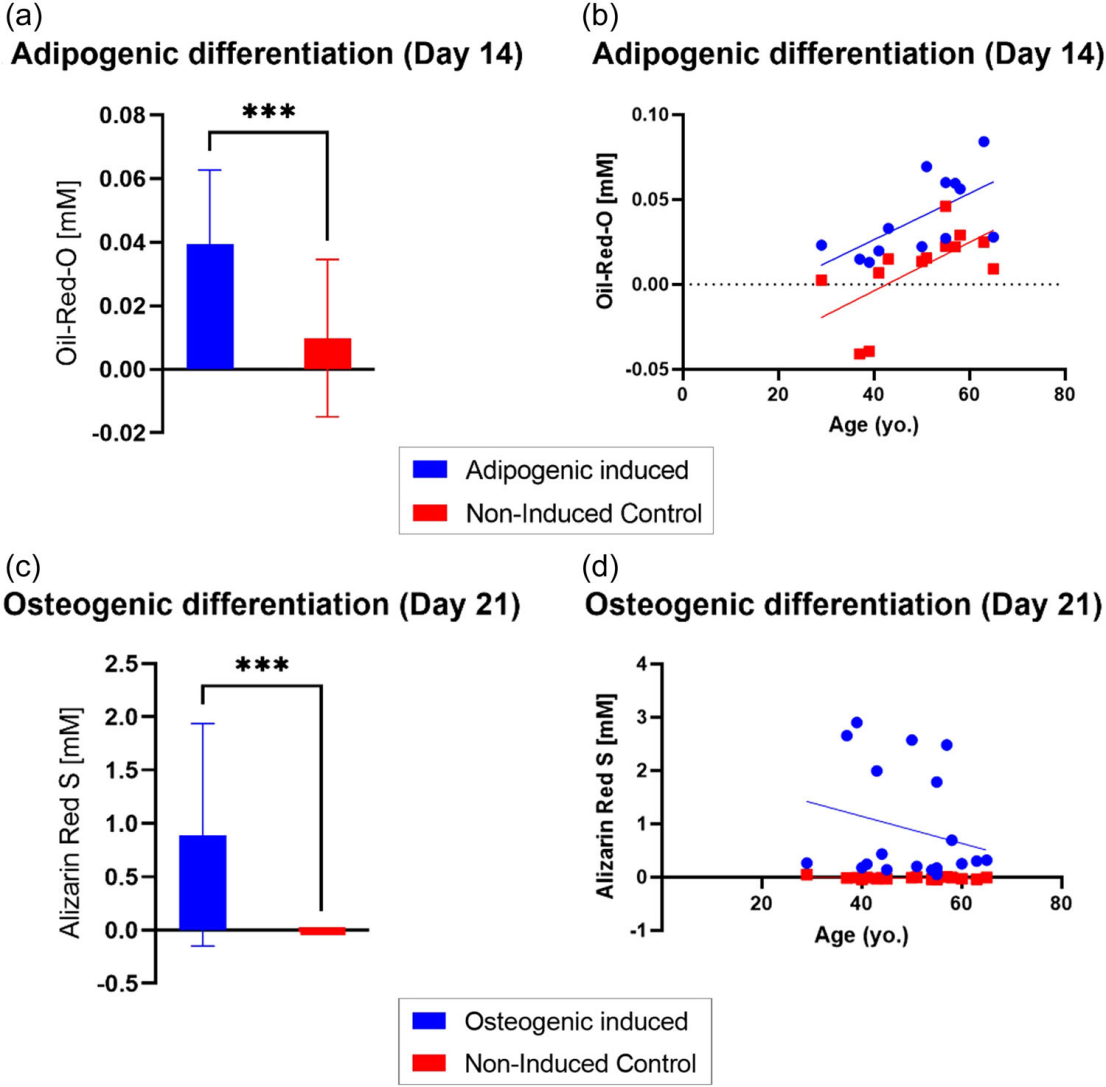
Adipogenic and osteogenic in vitro differentiation of MF‐ADSCs at passage 4. (a) Concentration of Oil‐Red‐O (absorbance measured at 490 nm) in the adipogenic induced cultures compared to the non‐induced controls at Day 14 of induction. Bar charts show mean and standard deviation. ***: *p* < 0.001 when analysed with Wilcoxon matched‐pair signed mark test. (b) Concentration of Oil‐Red‐O in the adipogenic induced cultures and the non‐induced cultures at Day 14 of induction as a function of patient age. (c) Concentration of Alizarin Red S (absorbance measured at 405 nm) in the osteogenic induced cultures compared to the non‐induced controls after 21 days of induction. Bar charts show mean and standard deviation. ***: *p* < 0.001 when analysed with Wilcoxon matched‐pair signed mark test. (d) Concentration of Alizarin Red S in the osteogenic induced cultures and the non‐induced cultures at Day 21 of induction as a function of patient age. ADSC, adipose tissue‐derived stem cell; MF, microfragmented; Yo., year old.

All samples (*n* = 13) showed adipogenic differentiation performance after 14 days of induction. The Oil‐Red‐O staining resulted in a significantly higher Oil‐Red‐O concentration in the adipogenic‐induced cultures compared to the non‐induced controls (*p* < 0.001) (Figure 3a). Furthermore, the concentration of Oil‐Red‐O staining significantly increased with increasing patient age for both adipogenic‐induced and non‐induced cultures (*p* = 0.021 and *p* = 0.022, respectively) (Figure 3b and Table 2).

**Table 2:** Overview of results.

Assay	*n*	Age (years) (mean (SD))	Slope	95% CI (slope)	*p* Value
Oil‐Red‐O					
Induced	13	49.5 (10.9)	0.001362	0.0003–0.0025	0.021
Non‐induced	13	49.5 (10.9)	0.001428	0.0002–0.0026	0.022
Alizarin Red S					
Induced	20	49.8 (9.58)	−0.02538	−0.778 to 0.027	0.322
Non‐induced	20	49.8 (9.58)	−0.0009473	−0.002 to 0.0001	0.066
qPCR					
CDKN2A	20	49.8 (9.58)	0.0001779	−0.0246 to 0.025	0.99
CDKN1A	20	49.8 (9.58)	−0.004773	−0.0278 to 0.0183	0.67
TP53	20	49.8 (9.58)	−0.001153	−0.0166 to 0.0143	0.88
GLB1	20	49.8 (9.58)	−0.002818	−0.0194 to 0.0137	0.72
IL6	20	49.8 (9.58)	0.006563	−0.0355 to 0.0486	0.75
CXCL8	20	49.8 (9.58)	0.001207	−0.062 to 0.0644	0.97
Flow cytometry					
p16	15	47.73 (9.11)	−0.1640	−0.4906 to 0.1626	0.3
p21	15	47.73 (9.11)	0.1367	−0.2551 to 0.5284	0.46
SA‐β‐GAL	19	49.74 (9.84)	0.07002	−0.1484 to 0.2885	0.51
EdU proliferation	20	49.8 (9.58)	0.2512	−0.66 to 0.3955	0.61

*Note*: slope refers to the linear regression models where data is presented as a function of patient age.

Abbreviations: CI, confidence interval; n, number of samples; SD, standard deviation.

All samples (*n* = 20) showed osteogenic differentiation performance after 21 days of induction. The Alizarin Red S concentration was significantly higher in the osteogenic‐induced cultures compared to the non‐induced controls (*p* < 0.001) (Figure 3c). No statistically significant difference was found in the concentration of Alizarin Red S staining as a function of patient age for the osteogenic induced or the non‐induced cultures (Figure 3d and Table 2).

### Generation of two positive controls of cellular senescent MF‐ADSCs

**Figure 4:**
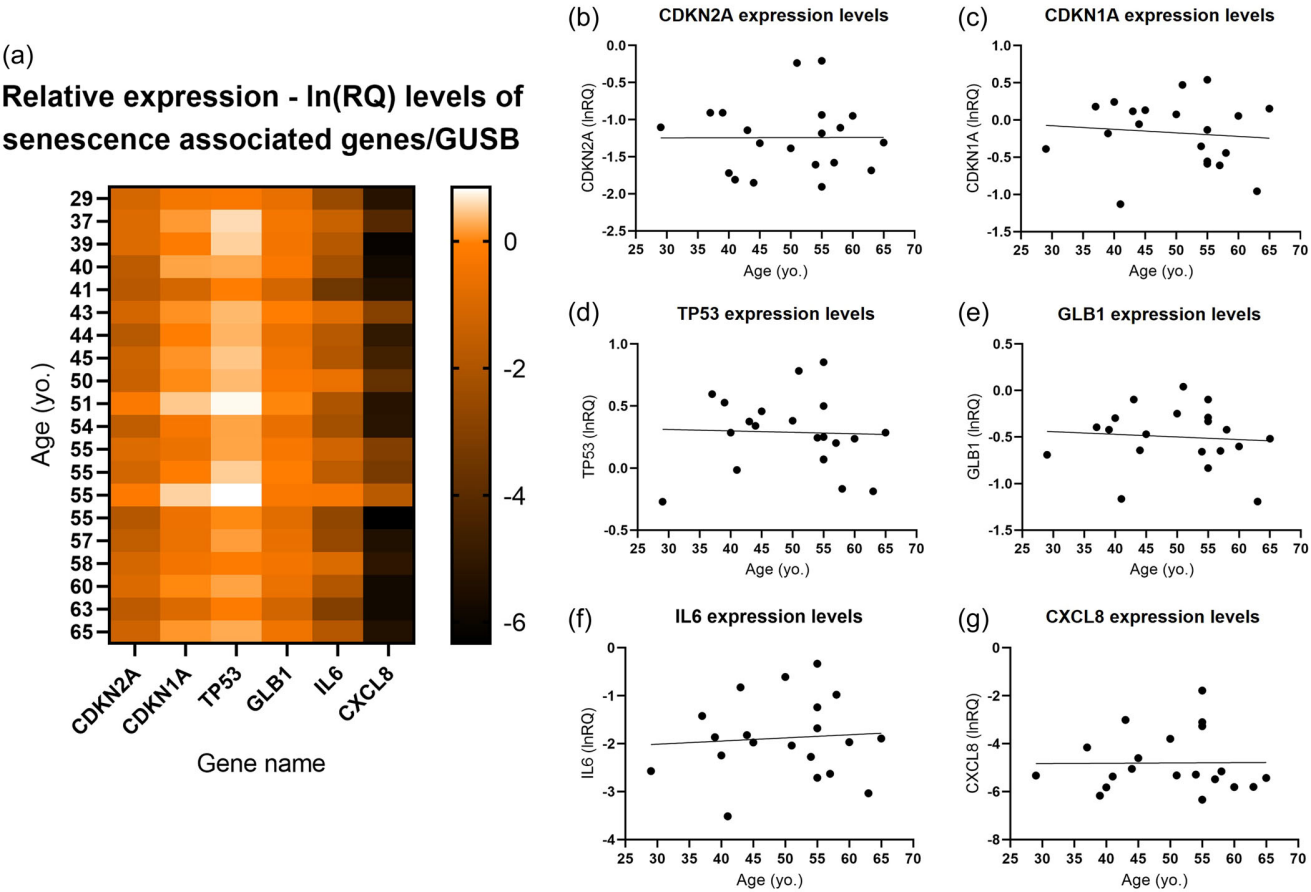
ln(RQ) steady‐state mRNA levels of senescence‐associated genes in MF‐ADSCs at passage 4 measured by qPCR. (a) Heatmap of gene expression as a function of patient age. The darker the colour of the heatmap, the lower the expression detected. (b) CDKN2A expression levels. (c) CDKN1A expression levels. (d) TP53 expression levels. (e) GLB1 expression levels. (f) IL6 expression levels. (g) CXCL8 expression levels. All values are calculated relative to a positive senescence control of MF‐ADSCs induced to undergo replicative senescence of which all ln(RQ) values equals 0. ADSC, adipose tissue‐derived stem cell; MF, microfragmented; Yo., year old.

**Figure 5:**
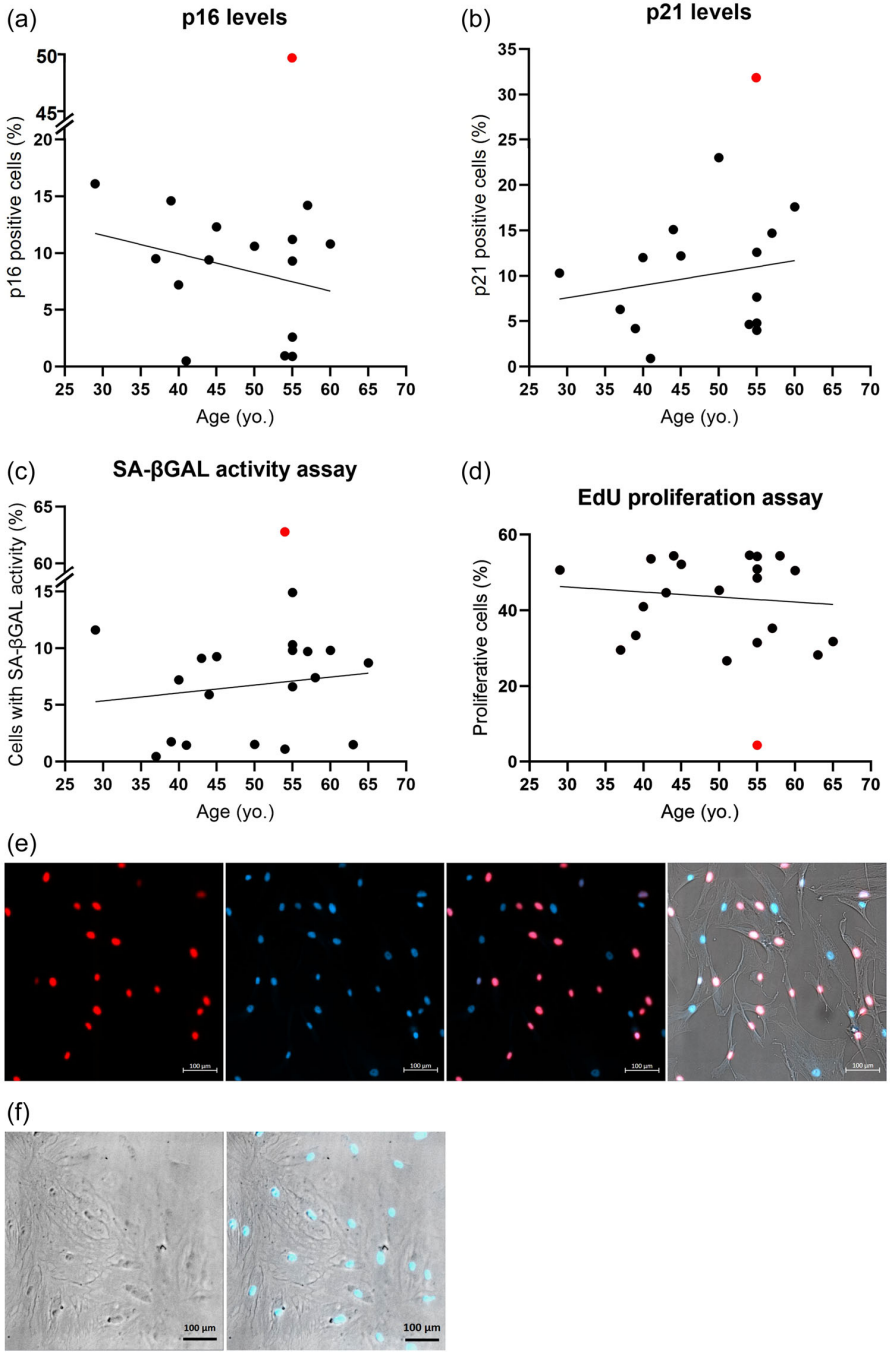
Percentage (%) of MF‐ADSCs at passage 4 with (a) intracellular senescence marker p16 and (b) p21, as a function of patient age after 1 h of spiking with p16 antibody Alexa fluor® 647 conjugate and p21 antibody Alexa fluor 488® conjugate. The positive senescent cell control showing stress‐induced premature senescence using H_2_O_2_ is represented by a red dot. (c) Percentage of MF‐ADSCs with SA‐β‐GAL activity as a function of patient age after 2 h of spiking with senescence dye at pH 6. The positive senescent cell control showing replicative senescence is represented by a red dot. (d) Percentage of proliferating MF‐ADSCs as a function of patient age. Proliferating cells were detected using a fluorescence microscope after spiking the cells with EdU for 24 h. Replicative senescent cells, as positive control, are represented by a red dot. Note: difference in y‐axis (a, c). Yo.: year old. (e) Representative images of MF‐ADSCs at passage 4 after 24 h of spiking with EdU. From the left; proliferating cell nuclei stained with Alexa Fluor 594 picolyl azide (red/pink) fluorescently detected with an Alexa Fluor 594 filter; nuclear cells counterstained with DAPI (blue) fluorescently detected with a DAPI filter; overlapping of the two stainings; overlapping of the two stainings and a bright field image (20×) showing the cells. (f) Brightfield (20×) and fluorescent overlapping (DAPI and Alexa 594) images of the positive senescence control of MF‐ADSCs induced to undergo replicative senescence after 24 h of spiking with EdU. ADSC, adipose tissue‐derived stem cell; MF, microfragmented.

MF‐ADSCs were induced to undergo replicative senescence, but unfortunately, not enough cells were obtained by this method to use as a positive senescent cell control for all assays. Replicative senescent cells can take months to generate. Instead, MF‐ADSCs that were subjected to H_2_O_2_ stress‐induced premature senescence, could be readily generated and were subsequently used as a positive senescence control for the intracellular senescence‐associated p16 + p21 proteins assay. Using both senescence induction methods, the MF‐ADSCs looked morphologically alike being enlarged, flattened, multinucleated and vacuolated. Senescent parameters for SA‐β‐GAL, steady‐state mRNA levels of senescence biomarkers, and cellular proliferation are shown in Figures 2, 4 and 5.

### Low steady‐state mRNA levels of IL6 and CXCL8 in MF‐ADSCs

Some samples exhibited elevated gene expression levels of TP53, CDKN1A and GLB1 gene expression, when compared to the positive senescence control. The gene expression levels of CDKN2A, IL6 and CXCL8 were generally low across all samples compared to the positive senescence control (Section 2, Figure 4a).

No significant difference was found in the gene expression levels for any of the biomarkers as a function of patient age (Figure 4b–g).

### Low protein levels of senescence‐associated p16 and p21 in MF‐ADSCs

Low protein levels of the intracellular senescence markers p16 and p21 were detected for all samples compared to the positive senescence control (*n* = 15). No statistically significant differences in the protein levels of the intracellular senescence markers p16 and p21 were identified in the samples as a function of patient age (p16; mean: 8.68%, SD: 5.18) (p21; mean: 10%, SD: 6.08) (Figure 5a,b). The positive senescence control (red dot) showed p16 and p21 levels of 49.8% and 33%, respectively (Figure 5a,b). As shown in Figure 2e, the cells positive for p16 (purple dots) and p21 (green dots) are relatively large in size and granulated.

Sixteen samples were analysed, but one sample was excluded due to a very low cell count (~1600 cells) and atypical‐looking histograms.

### Low levels of SA‐β‐GAL activity in MF‐ADSCs

Low levels of SA‐β‐GAL activity were detected across all samples (*n* = 19) (mean: 6.74%, SD: 4.26) after 2 h of spiking with senescence dye compared with the positive senescence control, which had SA‐β‐GAL activity in 62.8% of the cells. No statistically significant differences in the SA‐β‐GAL activity were found as a function of patient age (Figure 5c). As seen in Figure 2h, the cells positive for SA‐β‐GAL (shown in green) are large and granulated.

Twenty samples were analysed, but one sample was excluded due to a very low cell count and atypical‐looking histograms.

### Relatively high cellular proliferation capacity of MF‐ADSCs

Alexa 594 staining was detected in all EdU‐spiked cultures and none of the non‐EdU‐spiked negative controls (*n* = 20).

Relatively high levels of proliferating cells were detected across all samples (mean: 43.57%, SD: 10.29) after 24 h of spiking with EdU compared with the positive senescence control with 4.37% proliferating cells. No statistically significant difference in cellular proliferation was found as a function of patient age (Figure 5).

## DISCUSSION

### Analyses of the degree of cellular senescence

Low levels of cellular senescence were detected across all commonly used senescence assays from all patients included in this study compared to the positive senescence controls. The low level of cellular senescence from all included patients shows that microfragmentation is a gentle harvesting method that does not induce high levels of cellular senescence. Assessing levels of cellular senescence is important as injection of MF‐ADSCs with high levels of cellular senescence may diminish the regenerative capacity of the OA treatment.

In support of our findings, Ragni et al. [39] reported low levels of cellular senescence (≤5%) in freshly enzymatically processed MF AT from seven healthy donors aged 44 ± 6 years using a CD235a+ assay. However, Ragni et al. [39] only assessed senescence using this specific parameter and used enzymes for processing which differs from the current study where tissue explants were used to represent the non‐enzymatic processing of clinically used ADSCs from MF AT. No apparent difference was found between the level of senescence in samples from frozen MF AT and samples from MF AT directly cultivated as tissue explants [4].

**Figure 6:**
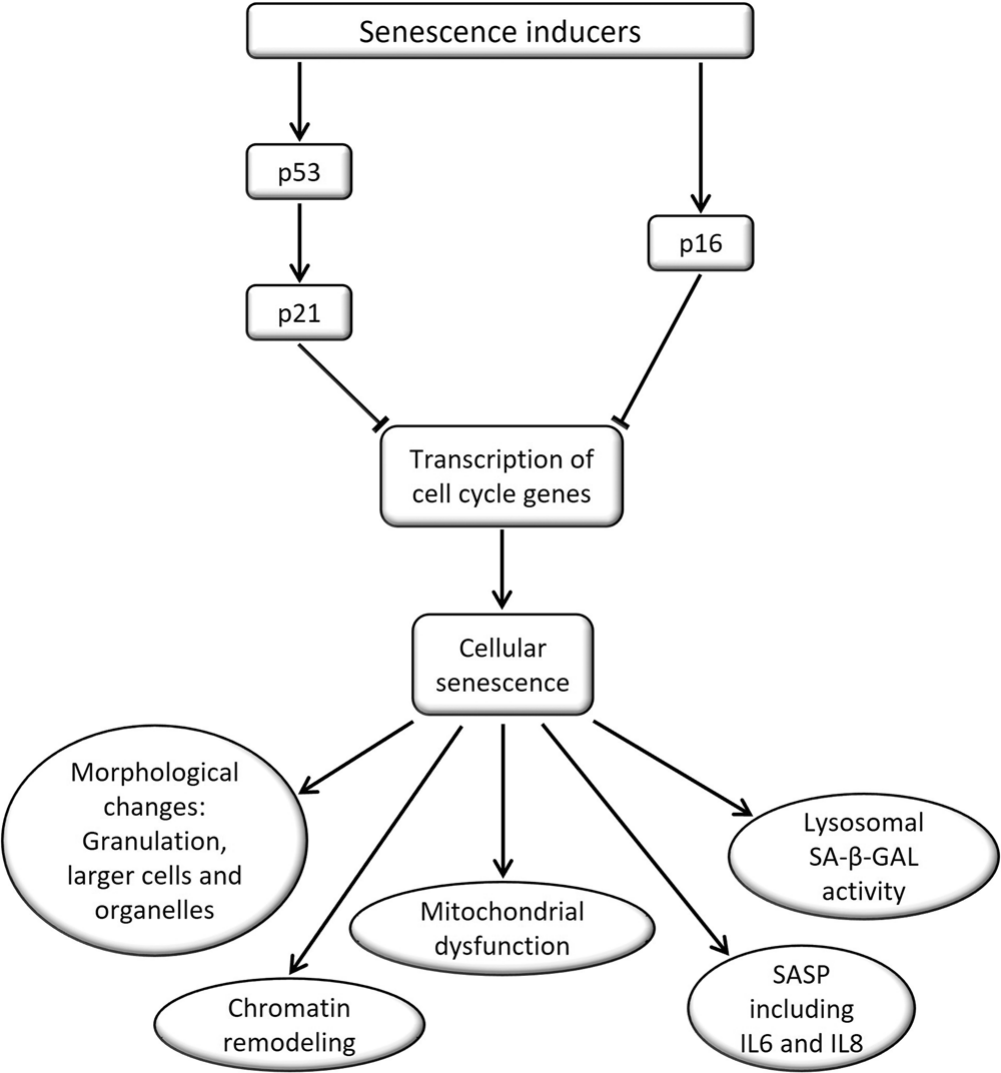
DNA damage, telomere shortening, disruption of chromatin organization, oncogene activation, oxidative stress, and mitochondrial dysfunction may accumulate in the cell over time. This may ultimately cause the cells to turn cancerous. To avoid this, the cell can either undergo apoptosis or become senescent. Continuous activation of the p53‐p21 or the p16 pathways inhibits transcription of cell cycle genes, irreversibly preventing cell proliferation, inducing cellular senescence and the associated characteristics. Based on Kumari and Jat [30] and Huang et al. [22].

In certain samples, the gene expression levels of CDKN1A, TP53 and GLB1 were either higher or nearly equivalent to those observed in the positive senescence control. The elevated gene expression observed for these markers may be attributed to a specific type of senescence present in the MF‐ADSCs subpopulations (Figure 6). DNA‐damage‐induced‐senescence is known to be associated with high levels of p21 and p53, encoded by the CDKN1A and TP53 genes, respectively [22, 30]. However, the biomarkers have additional functions, such as in apoptosis and cell cycle regulation, not directly associated with senescence, suggesting that the expression levels observed do not solely reflect a senescent state phenotype [22, 30]. A specific apoptosis assay was not performed in the current study as senescent cells are alive and metabolically active [30]. Assessing apoptosis was thus not within the scope of the current study aim. However, all samples were able to be expanded with high levels of live cells determined by trypan blue staining, as previously described [4].

GLB1 encodes a β‐GAL enzyme which exhibits activity across a broad pH range and is considered a housekeeping gene in mammalian cells. In senescent cells, an isoform of the enzyme is specifically active at pH 6 (SA‐ β‐GAL) [27, 47]. β‐GAL activity can thus be elevated in certain cell types or conditions unrelated to senescence, where GLB1 is involved in various cellular processes, and thus not completely specific to senescence. This could potentially explain the relatively elevated GLB1 gene expression observed. Nevertheless, the SA‐β‐GAL activity assay revealed low activity levels of the enzyme at pH 6 across all patients involved in the study, showing low levels of cellular senescence.

Qualitative assessment of SA‐β‐GAL activity is the gold standard for detection of cellular senescence [47]. In the present study, SA‐β‐GAL activity was assessed quantitatively and could therefore be used to compare the samples to each other. To our knowledge, this is the first study where SA‐β‐GAL activity has been shown to be high in senescent MF‐ADSCs, as observed in the positive senescence control. However, it is important to note that SA‐β‐GAL activity at pH 6 is not a definitive or exclusive marker for senescence and should therefore not be used solely to assess cellular senescence [33]. The enlarged and granulated morphology of the senescent control cells, as seen in the microscope and on flow cytometry (Figure 2e,h), is in conformity with previous studies on senescent cells [38].

Gene expression levels of the SASP markers, IL6 and CXCL8, were especially low compared to the positive senescence control. This indicates that IL6 and CXCL8 are better mRNA biomarkers of cellular senescence compared to the other biomarkers, as they are more in conformity with the low levels of cellular senescence seen across the other senescence‐associated assays. This finding is supported by Coppé et al. [13], who identified IL6 and CXCL8 as good gene markers of cellular senescence [13, 19, 41]. However, IL6 and CXCL8 are, like the other biomarkers, not necessarily senescence‐related even if they were to be translated and secreted, as they may also function in non‐senescence‐related inflammation [45].

There seems to be no correlation in the senescence characteristics when comparing the results from the same sample across the various assays. No clear pattern was identified between samples with a relatively high gene expression of CDKN2A, CDKN1A, TP53, or GLB1 and samples with a relatively high percentage of cells with intracellular p16 protein, intracellular p21 protein or SA‐β‐GAL activity, respectively. This heterogeneity can be attributed to the fact that each assay captures a snapshot of the cellular state at a particular moment, potentially leading to variations in the observed senescence characteristics. The mRNA must be translated into proteins, which need to be correctly folded, and some of them activated by phosphorylation or cleavage, before they can function in the senescent pathways. As such, TP53 is a transcription factor for CDKN1A but requires a phosphorylation to be stabilized, whereby TP53 and CDKN1A expression and p21 protein levels do not always correlate, as seen in the present study.

Collectively, these findings highlight the importance of evaluating a panel of multiple senescence‐associated biomarkers to enhance the comprehensive assessment of the results. In the current study, the level of cellular senescence was assessed both at gene expression, protein, protein activity (i.e., SA‐β‐GAL), and cellular functionality levels, which is more than commonly performed in previous studies on ADSCs [9, 10]. The overall low level of biomarkers of cellular senescence across the various assays corresponds well with the relatively high cellular proliferation capacity, the ability to expand all samples to ~70% confluency, and the successful adipogenic and osteogenic differentiation performance, demonstrating the functional potential of the stem cells.

### Effect of patient age on cellular senescence

Cellular senescence has been shown to decrease stem cell functionality with increasing donor age [7, 10]. In the current study, no correlation was found between senescence levels of MF‐ADSCs from OA patients aged 29–65 years and patient age for any of the assays typically used to quantify senescence. On the other hand, the senescence level of ADSCs from enzymatically processed AT was reported to increase with donor age in multiple previous studies [9, 10]. It should, however, be noted that these studies primarily looked at cell doublings, gene expression levels, and non‐quantitative SA‐β‐GAL activity, and importantly often included very young and/or very old donors, with typically few donors [9, 10]. Thus, it is reasonable to suggest that the age range of the patients in the current study (29–65 years) may not have been broad enough to detect an age‐related decline as reported by us and others on enzymatically processed ADSCs [3, 9, 10]. In support of our findings, ADSCs have been reported to be less affected by ageing than bone marrow‐derived stem cells when collected from aged osteoporotic patients and in a horse model [3, 9]. Moreover, most previous studies were performed on healthy donors, which is a more homogeneous study population than the present study population of OA patients. In an OA patient population, there could potentially be additional confounding factors contributing to the observed variance between samples and the absence of an age‐related correlation [9, 10]. It is possible, for instance, that young OA patients exhibit higher levels of senescence than young healthy donors, as senescent chondrocytes have been shown to play an important role in OA development [7, 10] where they accumulate and secrete multiple SASP factors contributing to the OA inflammatory environment [51], but more research is needed in this area.

Other parameters relevant for OA therapy, such as chondrogenic differentiation and extensive paracrine signalling, were not assessed as a function of patient age, but would be highly interesting for future studies. Nevertheless, the current study presents clinically important findings, as OA patients aged 29–65 years represent an important OA patient group.

### Stemness

Stemness was verified for all 20 samples. The adipogenic differentiation capacity improved with patient age for both the adipogenic‐induced and the non‐induced cultures. Other studies have found the same correlation, and it is generally accepted that ADSCs make an ‘adipogenic switch’ with ageing, whereby they are more prone to adipogenic differentiation compared to osteogenic differentiation [26]. In the current study, there was a trend towards lowered osteogenic differentiation performance with increasing age, but it was not supported statistically, and large individual variance was measured, which might be due to differences in immunophenotype composition.

Plastic adherent cells positive for the stem cell surface marker CD90 and negative for the blood cell surface marker CD45 and endothelial surface marker CD31 were characterized as stem cells [5, 38]. Additionally, the presence of clinically relevant MF‐ADSC subtypes was detected. Adventitial stem cells and pericytes from MF AT are known for having mesenchymal stromal cell characteristics in vitro [25] and have been demonstrated to regenerate the cartilage of OA joints [1, 25, 37]. Transitional pericytes are a recently detected cell type in MF AT positive for both CD34 and CD146 [38]. CD271+ ADSCs have been shown to enhance cartilage repair and decrease unwanted angiogenesis and are thus also of interest for the treatment of OA [28]. It should, however, be noted that the primary mechanistic treatment effects of unsorted ADSCs are through paracrine signalling [8].

### Methodological considerations

It would have been optimal to use the same positive senescence control for all assays. Unfortunately, this was not possible as inducing the MF‐ADSCs to undergo replicative senescence by repeated low seeding density was very time‐consuming and resulted in a low number of cells, which were very difficult to detach from the T75 flasks. These are classical features of cellular senescence [20]. MF‐ADSCs induced to undergo accelerated cellular senescence using H_2_O_2_ were employed as a positive control for the intracellular senescence‐associated p16 and p21 proteins assay, as these assays required more cells. Since p16 and p21 are intracellular markers, a methanol permeabilization was required for detection, which caused a notable loss of cells and resulted in numbers that were not sufficient for flow cytometry when using the replicative senescence method. The H_2_O_2_ method was thus selected as a positive control for the p16 and p21 assays, as it is an established and commonly used method to produce accelerated senescence of more cells with shared features of replicative senescence [16]. The H_2_O_2_ method was not applied as a positive control for all assays, as the replicative senescence method is the most representative of native cellular senescence, which will occur when the cells reach the Hayflicks limit [44]. The flow cytometric gatings of senescence markers were set based on the respective positive controls to account for autofluorescence of senescent cells primarily produced by lipofuscin, and thereby decrease false positives [36].

Assessing senescence to study multiple parameters required a relatively high number of cells. The assays were therefore performed at passage 4, which was the lowest passage number possible to have enough cells available to perform all assays. Cellular senescence was not measured at later passages, to provide data representative of the microfragmented point‐of‐care treatment given to OA patients. Passage 0 assays would, however, have been more representative. Culture and trypsinization of cells may affect the cells and enhance cellular senescence, as previously reported around passage 7 [15, 35], but this is believed to be of minor importance to the reported data as all samples were able to be expanded, showed stemness, and negligible levels of senescence. A maximum confluency of 70% was selected to avoid cell‐contact inhibition that could be a potential risk factor to initiate senescence [21].

Samples from healthy donors were not included in the current study, and it is unclear if even lower levels of cellular senescence may benefit the treatment effect.

## CONCLUSIONS

No significant difference in the level of typical cellular senescence markers was identified as a function of patient age when analysing MF‐ADSCs from abdominal AT from knee OA patients aged 29–65 years.

Low levels of several cellular senescence biomarkers were detected among MF‐ADSCs in all samples derived from abdominal AT from knee OA patients compared to the positive senescence controls.

Autologous MF‐ADSCs may be used in clinical trials of knee OA for patients aged 29–65 years, at least until passage 4, as they show stemness potential and negligible senescence in vitro.

## AUTHOR CONTRIBUTIONS

Jasmin Bagge, Freja Aabæk Hammer, Kristoffer Weisskirchner Barfod, Per Hölmich and Jan O. Nehlin were responsible for the study design. Jasmin Bagge, Per Hölmich, Kristoffer Weisskirchner Barfod and Jan O. Nehlin were responsible for obtaining funding. Kristoffer Weisskirchner Barfod, Jasmin Bagge, Per Hölmich, Lisbet Rosenkrantz Hölmich and Lars Blønd were responsible for the conception and harvest of microfragmented adipose tissue. Acquisition of data, data analysis, and interpretation, and the first draft of the manuscript was done by Freja Aabæk Hammer and Jasmin Bagge. Jan O. Nehlin provided expert assistance with the positive senescence controls. Kilian Vomstein provided assistance with single‐ and multicolour flow cytometry set‐up and assessment of data. All authors were involved in the critical revision of the manuscript. All authors read and approved the final manuscript.

## CONFLICT OF INTEREST STATEMENT

The authors declare no conflict of interest.

## ETHICS STATEMENT

Collection, molecular analysis, and biobanking of the cells were approved by the Danish National Committee on Health Research Ethics (H‐18013145) and the Danish Data Protection Agency (VD‐2018‐141).

## Data Availability

The data sets used and/or analysed during the current study are available from the corresponding author on reasonable request.
